# The Great Masquerader: Recurrent Ischemic Strokes Secondary to Meningovascular Syphilis in a Man With Cardiovascular Stroke Risk Factors

**DOI:** 10.7759/cureus.56330

**Published:** 2024-03-17

**Authors:** Octavio Carranza, Sadia Waheed, Fawad Yousuf

**Affiliations:** 1 Neurology, Florida Atlantic University Charles E. Schmidt College of Medicine, Boca Raton, USA

**Keywords:** vascular neurology, atypical stroke, cerebro-vascular accident (stroke), tertiary syphilis, meningovascular neurosyphilis

## Abstract

A male in his 60s with stroke risk factors presented with confusion and word-finding difficulties. He was diagnosed with acute ischemic stroke in the right basal ganglia. He was started on secondary stroke prevention measures including dual antiplatelet therapy and a high-dose statin. A highly reactive rapid plasma reagin (RPR) was performed as part of the workup and found to be positive. Follow-up fluorescent treponemal antibody absorption (TPA) test was also positive, confirming a diagnosis of syphilis. He was discharged home with a scheduled course of antibiotic treatment for tertiary syphilis but returned due to a new episode of transient facial paralysis. Further workup and physical exam findings revealed the patient had neurosyphilis. He was started on the appropriate antibiotic therapy, which significantly improved his confusion and prevented new episodes of stroke.

## Introduction

The prevalence of syphilis in the US is 9.5 cases per 100,000. About 3.5% of these patients develop neurological manifestations. It was previously thought that neurosyphilis presented only with advanced disease, given the natural evolution of the disease and its temporal and ordinal sequence of primary, secondary, and tertiary stages. It is now widely accepted that the treponeme can invade the central nervous system within days of infection and remain asymptomatic for as much as two years [1].

## Case presentation

A male in his 60s presented to the emergency department after experiencing progressive confusion, word-finding difficulties, and “brain fog.” 

Past medical history was significant for diabetes mellitus, coronary artery disease with recent drug-eluting stent placement, and an ischemic stroke four months prior to presentation. The previous stroke left the patient with residual visual field changes as he was unable to receive timely treatment.

On examination the patient was unable to identify the year. He had difficulty recalling three words after five minutes, had word-finding difficulty, and struggled to engage in conversation when asked questions. His attention span, concentration, fund of knowledge, and awareness of past events were intact. He was not dysarthric and naming, repetition, and comprehension were normal. Cranial nerve examination revealed a left superior quadrantanopia on visual field examination. His strength and sensory exam were normal, and examination of his gait revealed unsteadiness on tandem walking without a characteristic pattern.

**Figure 1:**
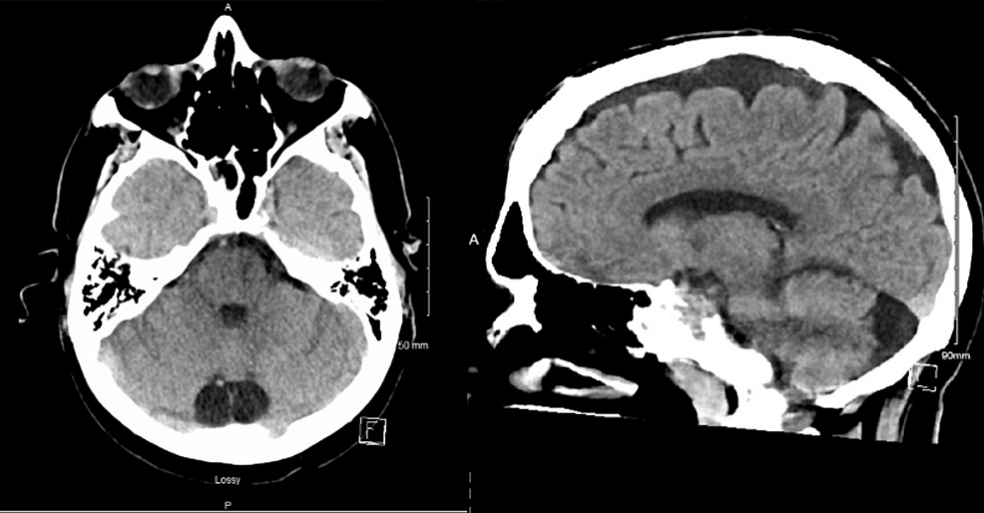
CT Brain without contrast with no signs of acute intracranial hemorrhage, mass effect, midline shift or herniation and an incidental finding on cysterna magna.

CT scan of the brain was performed and showed no evidence of intracranial pathology (Figure 1). Urinalysis showed significant pyuria and antibiotics were initiated. The patient was admitted to the hospital for acute encephalopathy secondary to a urinary tract infection.

**Figure 2:**
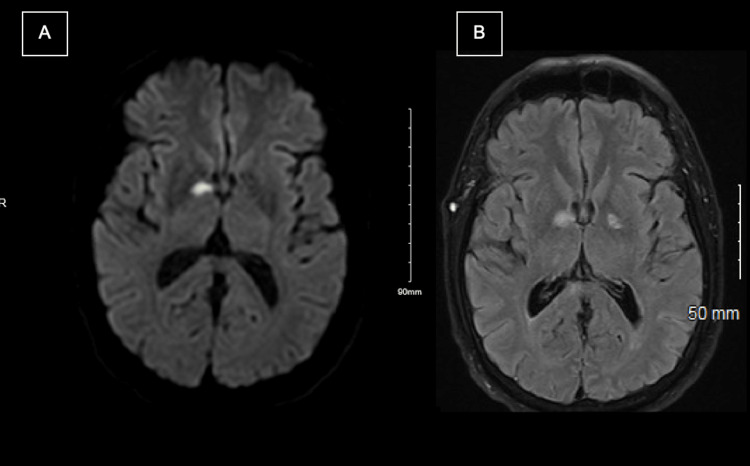
MRI Brain without contrast: A) DWI sequence with an evident focal restricted diffusion in the right basal ganglia; B) T2/FLAIR sequence showing a hyperintense lesion in the same region consistent with subacute right basal ganglia infarction. DWI: Diffusion-weighted imaging FLAIR: Fluid attenuated inversion recovery

MRI of the brain without contrast showed an area of focal restricted diffusion in the right globus pallidus region consistent with a subacute infarct of the right basal ganglia. There was also evidence of a remote hemorrhagic infarct of the right occipital lobe and scattered foci of periventricular, subcortical and deep white matter hyperintensities in T2-fluid attenuated inversion recovery (FLAIR) which were signs of multiple previous strokes throughout his brain (Figure 2).

**Figure 3:**
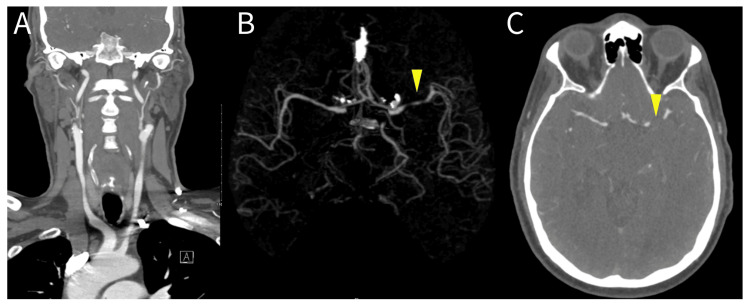
CT Angiogram of the head and neck: A) Atheromatous changes in the internal carotid arteries without associated stenosis; B & C) 3D reconstruction of CT Angiogram and axial image of CT Angiogram of the brain. Yellow arrowheads point towards the patient's left MCA stenosis. MCA: middle cerebral artery

CT angiography of the head and neck demonstrated patent common carotid and vertebral arteries without hemodynamically significant stenosis and high-grade stenosis without complete occlusion of the left middle cerebral artery (MCA) in the M1 and M2 segments (Figure 3). Due to these findings, the patient was started on dual-antiplatelet therapy and high-dose statin for secondary stroke prevention measures.

Rapid plasma reagin (RPR) testing was performed as part of the initial workup for confusion and was reported as reactive with a titer of 1:256. The patient denied any history of genital ulcers or activities of high risk but reported unintentional weight loss of 40 lbs over the last four months. Follow-up fluorescent treponemal antibody absorption test (FTA-ABS) was positive, confirming a diagnosis of syphilis.

A confirmatory lumbar puncture was planned to rule out neurosyphilis. Due to the patient's initiation of clopidogrel for stroke prevention and the requirement of a five-day waiting period per institutional guidelines, the lumbar puncture could not be completed immediately as the benefits of obtaining a cerebrospinal fluid (CSF) sample were outweighed by the risk of discontinuing dual antiplatelet therapy (DAPT) after a recent stroke.

The possibility of recurrent strokes from syphilis was considered, however, because the diagnosis of neurosyphilis could not be made at the time and the stroke workup showed the presence of intracranial atherosclerosis, he was prescribed three weekly doses of 2.4 million units of penicillin, instead of the penicillin regimen for neurosyphilis (18-24 million units/10-14 days). 

The patient continued taking DAPT and high-dose statin and followed up with the Infectious Disease outpatient clinic for the next three weeks without any new symptoms. On the third week after his discharge, he had a new episode of transitory facial droop that prompted him to return to our hospital. The facial droop had resolved upon arrival to our emergency room and his neurological examination was unchanged from his prior admission. Repeat MRI of the brain was negative for a new stroke but revealed acute scattered brain petechial hemorrhages. A lumbar puncture was performed during the second hospitalization, which revealed positive venereal disease research laboratory (VDRL) and positive treponemal antibodies in CSF to support a diagnosis of neurosyphilis. The patient was prescribed the appropriate dose and course of antibiotics and discharged home independently.

The patient continued to follow up with the infectious disease specialist as an outpatient. Following treatment, his confusion improved significantly and he had no new or recurrent stroke-like symptoms. The patient continued to have baseline peripheral vision deficits and word-finding difficulties from his previous strokes.

## Discussion

The differential diagnosis of confusional syndrome is broad and includes primary central nervous system (CNS) structural lesions, metabolic derangements, hormonal imbalances, medication side effects, and illegal drugs, cerebral or systemic infections, psychiatric disorders, and degenerative diseases of the brain.

Confusion and word-finding difficulty in this patient were initially thought to be secondary to acute encephalopathy after he was found to have a urinary tract infection. Acute encephalopathy is mainly characterized by impaired attention and concentration, sometimes associated with fluctuation in the state of consciousness due to a disturbance of the reticular activating system in the brain. While there are many variable manifestations across its different etiologies, acute encephalopathy should be seen as a diagnosis of exclusion.

The patient in our case had no impairment in his attention or concentration. His chief complaints were his anterograde memory and lexical retrieval difficulties. Due to his recent ischemic stroke with sequela of a left superior quadrantanopia, a stroke work-up was ordered. Other causes of confusion were also explored with a urine drug screen, a basic metabolic panel, ethanol in serum, vitamin B12 and thiamine levels, thyroid stimulating hormone (TSH), and RPR.

Brain imaging revealed the presence of a right basal ganglia subacute infarction and a remote infarction in the right occipital lobe. The patient's left homonymous superior quadrantanopia was explained by previous damage to the contralateral inferior part of the posterior visual pathway in either the temporal Meyer loop or the inferior calcarine fissure. Because only half of the macula is required for visual acuity, this patient did not manifest with any decreased vision [2]. His lexical retrieval difficulties however were explained by the new stroke. Even though the basal nuclei (putamen, caudate and globus pallidus) do not actively participate in language production, they are involved in linguistic processing [3].

After being found to have a new infarction four months after a previous one, he was evaluated for cardioembolic and atherothrombotic mechanisms of stroke. Vessel imaging revealed significant intracranial atherosclerosis and he was prescribed a statin and dual-antiplatelet therapy for recurrent stroke prevention.

There are five clinical syndromes of neurosyphilis recognized: 1) early asymptomatic neurosyphilis, 2) syphilitic meningitis, 3) meningovascular syphilis, 4) general paresis and 5) tabes dorsalis [4].

Diagnosis of syphilis can go undetected if the primary lesions are in hard-to-view areas such as the rectum or cervix. Other signs, like the pathognomonic rash, can be mistaken for other conditions, such as viral exanthemas or drug reactions. For this reason, the diagnosis is usually based on supportive laboratory findings [5].

Detection of Treponema on dark field microscopy or PCR of T. pallidum from an active lesion exudate can give a definitive diagnosis of syphilis. More commonly, the workup of syphilis includes non-treponemal (RPR and VDRL which detect antibodies and nonspecific antigens, like cardiolipin, present on the spirochete's wall) or treponemal (FTA-ABS which are specific for T. pallidum) studies. Traditionally, the diagnostic algorithm for syphilis includes a positive non-treponemal test and a confirmatory nontreponemal test.

For neurosyphilis however, a cerebrospinal fluid analysis is required in addition to positive serologic testing. A definitive diagnosis of central nervous system involvement must be confirmed with a reactive VRDL in the CSF [6].

Meningovascular syphilis (MVS) is usually a manifestation of tertiary syphilis. In MVS, there is obliteration of the blood vessels in the leptomeninges, brain, and spinal cord secondary to chronic endarteritis, which eventually leads to neural tissue infarction. This pathological finding was previously named Heubner's arteritis, when medium to large-sized vessels are involved, and Nissl-Alzheimer's arteritis when small-sized arteries are involved [7].

While syphilis was a common cause for stroke before widespread antibiotic use, it is not identified correctly in almost 80% of cases. Strokes due to syphilis most commonly involve the MCA and less commonly the basilar or anterior spinal arteries. [8].

Some studies have evaluated the value of doing serological screening in stroke patients; one study in particular found that 4% of recurrent strokes or transient ischemic attacks (TIA) can be due to syphilis [9]. Syphilis does not necessarily need to progress to the final stages of the disease to affect the nervous system. Thus, patients with a history of other STDs, HIV, and recurrent strokes, particularly in young patients, might benefit from routine screening with non-treponemal antibodies [10].

Treatment of neurosyphilis involves a course of parenteral penicillin G, 18-24 million units per day for 10-14 days, given as 3-4 million units every four hours or as continuous intravenous infusions. While there is some evidence for treatment with ceftriaxone and doxycycline which typically work for other types of spirochetal infections, treatment with penicillin is still preferred. In the setting of a penicillin allergy, desensitization against the drug is recommended [11]. After treatment is complete, RPR titers should be monitored until they return to normal range.

## Conclusions

While uncommon, syphilis remains prevalent in the United States. Neurological involvement can occur at any stage of the disease. Meningovascular syphilis, a subtype of neurosyphilis, can cause recurrent ischemic strokes.

Definitive diagnosis of neurosyphilis requires a reactive VDRL test in CSF. It is important to always keep neurosyphilis as a diagnostic differential in patients with recurrent strokes, even if there are no pertinent history or risk factors reported. The antibiotic therapy for both syphilis and neurosyphilis continues to be Penicillin G however, the dose for neurosyphilis is higher across 10-14 days.
